# Self-Standards and Self-Discrepancies. A Structural Model of Self-Knowledge

**DOI:** 10.1007/s12144-013-9203-4

**Published:** 2014-01-24

**Authors:** Waclaw Bak

**Affiliations:** Institute of Psychology, Department of Personality Psychology, The John Paul II Catholic University of Lublin, Al. Raclawickie 14, 20-950 Lublin, Poland

**Keywords:** Self-standards, Self-discrepancies, Can self, Attainability of standards, CFA

## Abstract

A model of self-knowledge is proposed which summarizes and integrates a few distinctions concerning self-standards and related self-discrepancies. Four types of self-standards are distinguished (i.e. ideal, ought, undesired and forbidden selves) and a hierarchical organization of these standards is postulated. There is a basic contrast between positive and negative standards at the higher level of the hierarchy, whereas Higgins’ distinction between ideals and oughts is found at the lower level. Every self-standard is analyzed in terms of two types of self-discrepancies. Many previous studies explored discrepancies between self-standards and the actual self, i.e. the perceived actualization of standards. The present study proposed that discrepancies between self-standards and the can self are a second type of discrepancy that should be included in structural models of self-knowledge. The can self consists of self-beliefs referring to capabilities and potentials; thus, this additional type of discrepancy reflects the perceived attainability of standards. Consequently, the present study explored a set of eight self-discrepancies, i.e. both the perceived actualization and the attainability of four self-standards. In order to assess the intercorrelations among these eight self-discrepancies, participants (*N* = 404) completed a newly developed online measure. CFA modeling confirmed the postulated two-level hierarchy of self-standards. The reasonability of including discrepancies between self-standards and the can self in the structural model of self-knowledge was also confirmed.

Subjective beliefs about oneself play an important role in the processes of self-regulation. Setting goals and monitoring progress towards their achievement requires self-knowledge, referring to some standard (e.g. the ideal self), as well as an assessment of the degree of actualization of that standard. This idea is clearly described by Duval and Wicklund (1972) in their objective self-awareness theory (see also Silvia and Duval 2001). They conceptualized self-regulation as a process of dealing with the perceived discrepancy between the actual self and self-standards. When attention is focused on the self, one compares actual self-view with a particular standard. Perceived discrepancy between self and standard results in negative affect, which induces various self-regulatory strategies.

The aim of the present paper is to construct and test a structural model of self-beliefs that may be useful in analyzing self-regulation processes, though self-regulation per se is not directly studied here. This research is focused on self-knowledge – i.e., subjective self-perception and beliefs about oneself. The behavioral aspects of self-regulation are not measured and, consequently, they are not included in the analyses. However, based on theoretical models of self-regulation (e.g., Carver and Scheier 1998; Duval and Wicklund 1972; Higgins 1997), hypothetical links between self-knowledge and behavior are formulated throughout the paper. Assuming the central role of self-standards in self-regulation, a structural model of self-knowledge is proposed, which focuses on (a) detailed description of self-standards with a proposal of their taxonomy and hierarchy as well as on (b) the structure of self-discrepancies – i.e., the relations between self-standards and other aspects of self-beliefs.

## Self-Standards

Self standards are preferential self-beliefs (as opposed to descriptive and evaluative ones), which consist of self-imposed criteria for judging oneself (Morris and Kanfer 1995). They are self-views that refer to some desired or undesired end-states. The concept of self-standards can be seen in the broader context of possible selves theory (Markus and Nurius 1986). In contrast to the actual (current) self, self-standards are possible selves^1^ and, as such, they refer to imagined future states of the self that may be actualized one day but may also never be reached. Markus and Nurius (1986, p. 954) distinguished between the possible selves “we would very much like to become” and the possible selves “we are afraid of becoming.” The positive future potentials are called hoped-for selves, while the negative, unwanted possibilities are called feared selves. According to this classic approach, although the possible selves are cognitive structures (i.e., they refer to self-perception, not to behavior per se), they operate as incentives for future behavior (e.g., Freeman et al. 2001; Robinson et al. 2003; Sobh and Martin 2008). Recent analyses specified the conditions for possible selves to be effective regulators of behavior (Norman and Aron 2003; Oyserman and James 2009) and the mechanisms by which the process operates. Hoyle and Sherrill (2006) proposed that possible selves do not directly influence behavior but rather function as sources of standards for self-regulation – “standards against which current self-representation is compared and with which it is reconciled through behavior” (p. 1687) (see also vanDellen and Hoyle 2008). Hoped-for selves function as positive standards while feared selves function as negative standards.

The idea that there are both positive and negative standards and that they cooperate in the process of self-regulation was also clearly expressed by Carver and Scheier (1998) in their control theory of approach and avoidance. They distinguished between two systems of regulating behavior. The approach system has a positive standard as the reference point and the ultimate goal of the system is to move the actual self as close as possible to the positive standard – that is, to minimize the discrepancy between the self and the standard. The avoidance system, in contrast, has a negative standard as the reference point and the ultimate goal of the system is to move the actual self as far as possible away from the negative standard – that is, to maximize the discrepancy between the self and the standard. The two systems represent independent, though functionally interconnected, processes of self-regulation (Carver et al. 1999; Carver and Scheier 1998; Woodman and Hemmings 2008). Carver (2006) argued that approach and avoidance are the very basic behavioral tendencies, which are closely related to temperamental dimensions of BAS (behavioral activation system) and BIS (behavioral inhibition system), respectively, and managed by distinct neural structures. Assuming that the approach-avoidance distinction is so fundamental for the analyses of self-regulatory processes, the present study postulates that the distinction between positive and negative standards (i.e., reference points for approach and avoidance, respectively) should also be seen at the very basic level in the structural models of self-knowledge.

However, besides this general distinction between positive and negative self-standards, other differentiations can be found in the literature. Among the most important ones, there is the distinction between an ideal self and an ought self as proposed by Higgins (1987) in his self-discrepancy theory. The ideal self is the representation of the attributes that one would ideally like to possess – the representation of one’s hopes, aspirations, and wishes. The ought self represents the attributes that one believes one should possess – that is, one’s sense of duty, obligation, and responsibility. The two self-standards are responsible for vulnerability to different negative emotions – dejection-related and agitation-related emotions, respectively (Higgins 1987). They also operate as bases for two different regulatory foci – the ideal self for promotion regulatory focus and the ought self for prevention regulatory focus (Higgins 1997).

Despite the differences, however, both the ideal self and the ought self refer to some desired end-states that are to be approached. Hence, although there is a difference between ideal-based and ought-based desirability, both the ideal self and the ought self represent positive standards. We can say, then, that Higgins’ differentiation is, in essence, a more detailed description of the general category of positive standards. A question arises of whether negative standards can be differentiated in a similar way. If the ideal self and the ought self are two types of positive, approach-related standards, an analogous distinction between “un-ideal” and “un-ought” selves as two types of negative, avoidance-related standards seems reasonable. The literature, however, is less explicit about it and the latter distinction needs more direct formulation.

The concept of *undesired self* fits the meaning of un-ideal self well. Ogilvie (1987) defined the undesired self as the opposite of the ideal self and operationalized it as an answer to the question: “How I hope to never be?” He argued that the undesired self is an important self-standard whose regulatory significance has been erroneously neglected in most analyses of emotional and motivational consequences of self-knowledge. The discrepancy between the undesired self and the actual self seems to be even more important than the discrepancy between the ideal self and the actual self in predicting life satisfaction (Ogilvie 1987; Ogilvie and Clark 1992), self-esteem (Endo 1992), and negative affect (Cheung 1997; Heppen and Ogilvie 2003; Ogilvie et al. 2008; Phillips et al. 2007). Moreover, the correlations between these two types of self-discrepancies (i.e., the perceived actualization of the undesired self and the perceived actualization of the ideal self) are weak and insignificant (Heppen and Ogilvie 2003; Ogilvie and Clark 1992). This suggests that the undesired self is not a simple reverse of the ideal self but rather an independent self-standard.

In contrast to the well-established concept of the undesired self, the conceptualization of the negative counterpart of the ought self has not been so clearly formulated. There is an example of the term *feared self* being used in this un-ought sense (Carver et al. 1999). Nevertheless, the basic definition of the feared self proposed by Markus and Nurius (1986) does not specify it in this way. In most studies referring to this term (e.g. Sobh and Martin 2008; Vignoles et al. 2008; Woodman and Hemmings 2008) it is not clear whether the feared self is the possible self that a person does not want to become (i.e. the un-ideal self) or rather that which a person believes they should not become (i.e. the un-ought self). Usually, the term feared self is used in a general sense, referring to negative standards, without further differentiation being made. Despite this lack of explicit formulation in the existing literature, the distinction between ideal and ought aspects of negative self-standards seems to be a legitimate element in the typology of self-standards.

Summing up, there are two distinctions within the field of self-standards: (a) between positive and negative standards and (b) between ideals and oughts. It is proposed here that these distinctions are not competing ones, but rather that they can be combined, resulting in the following two-level typology of self-standards:

1. Positive standards – reference points for approach-related self-regulatory processes
  1.1. The ideal self – the representation of the attributes that one would like to possess, reflecting one’s hopes, aspirations, and wishes.
  1.2. The ought self – the representation of the attributes that one believes one should possess, reflecting one’s sense of duty, obligation, and responsibility.
2. Negative standards – reference points for avoidance-related self-regulatory processes
  2.1. The undesired self – the representation of the attributes that one would not like to possess – i.e., the negative counterpart of the ideal self.
  2.2. The forbidden self – the representation of the attributes that one believes one should not possess – i.e., the negative counterpart of the ought self.^2^

The proposed typology distinguishes between ideal and ought aspects of both positive and negative self-standards. It is hypothesized here that self-standards are hierarchically organized – i.e., the ideal-ought distinction is nested within the higher-level (i.e., more basic) positive–negative distinction. The general category of positive standards is postulated to be superior to the distinction between the ideal self and the ought self. Analogically, the general category of negative standards is postulated to be superior to the distinction between the undesired self and the forbidden self. The verification of this postulated hierarchy of self-standards is one of the two main objectives of the present study.

## Self-Discrepancies

Self-standards play an important role in the processes of self-regulation. What is crucial, however, is not the mere content of a particular standard but the relations between self-standards and other aspects of self-knowledge. These relations can be described in terms of self-discrepancy, which is defined as the degree of dissimilarity between two given aspects of self-knowledge. Among different self-discrepancies, those reflecting the relations between self-standards and the actual self have received the most theoretical and empirical attention (e.g., Amico et al. 2004; Phillips and Silvia 2010; Vangronsveld et al. 2011; Wasylkiw et al. 2010). Self-discrepancies of this type reflect the perceived degree of actualization of standards and, as such, play an important role in the emotional, motivational, and behavioral aspects of self-regulation. According to this view, self-regulation is the process of reducing the discrepancies between the actual self and positive standards and enlarging the discrepancies between the actual self and negative standards (e.g., Carver and Scheier 1998).

In the present paper I argue that, apart from the undoubtedly important discrepancies between self-standards and the actual self, there are self-discrepancies of another kind that should be included in models of self-regulation. They are discrepancies between self-standards and *the can self.* Higgins et al. (1990) defined the can self as the element of self-knowledge referring to one’s capabilities and potentials. It is one’s representation of self attributes that are expected to be actualized in the future – i.e., the attributes that one believes she/he can possess even if they have not been actualized yet (Higgins et al. 1990, p. 171). Thus, discrepancies between self-standards and the can self reflect the perceived attainability of standards. In the case of positive standards, the smaller the discrepancy, the more firmly one is convinced that the desired end-state will be attained. The opposite applies to negative standards: the smaller the discrepancy, the higher the perceived risk of unwanted possibilities becoming actualized.

It seems reasonable that the emotional, motivational, and behavioral aspects of self-regulation depend not only on the degree to which standards are perceived as already realized, but also on the expectations that standards will be realized in the future. In most cases, those two types of self-discrepancies are probably related. Usually, the greater the distance between a person’s actual self-view and the desired end-state, the weaker the belief that this discrepancy can be reduced. Conversely, the closer the actual self is to an individual’s standard, the more confident the individual is that he or she is able to actualize the standard. It is possible, however, that the perceived actualization of standards and their perceived attainability operate separately. We can think of a situation in which a person perceives themselves as highly different form their ideals (large actual-ideal discrepancy) but at the same time is strongly convinced that they have all the capabilities and resources that are needed to reach this desired goal in the future. On the other hand, we can imagine the opposite situation: one in which a person is quite close to his or her ideal goal but at the same time does not believe that he or she is capable of reaching it.

The perceived attainability of standards, then, appears to be an important aspect of self-beliefs: one that should be included in the structure of self-discrepancies as one of its components. The idea that self-knowledge regarding one’s potentials and capabilities plays an important role in self-regulation is not new in psychology. It has been clearly expressed by Bandura (e.g., 2001) and widely studied within the self-efficacy paradigm (e.g., Endler et al. 2001; Flett et al. 2011; Maddux and Volkmann 2010; Oleś et al. 2013). Although there is striking similarity between the concepts of self-efficacy and can self, certain important differences between the approach employed in the present study and the classic self-efficacy paradigm should be emphasized. First, self-efficacy beliefs are conceptualized as agentic capability – a belief that one can perform a given activity as the agent (Bandura 2001). This agentic aspect, however, is not so central in the present study as it is in the case of self-efficacy. The can self as a specific aspect of self-knowledge refers to the belief that a particular self-view can possibly become the actual self in the future, regardless of the degree of agency that the subject attributes to himself or herself.

Second, the aim of the present study is to place the can self in the framework of self-discrepancy theory along with discrepancies between self-standards and the actual self. There is, to my knowledge, only one published study that addresses this problem directly. Higgins et al. (1990) provided an interesting theoretical analysis and preliminary empirical data showing that the discrepancy between the ideal self and the can self moderates the regulatory functions of the discrepancy between the ideal self and the actual self. They postulated that discrepancies should be considered in terms of a holistic pattern rather than viewed separately. The perceived attainability of standards seems to be an important component of such a holistic model.

## The Present Study

The present study started with a model that, synthesizing all the above-mentioned aspects of self-knowledge, distinguishes four different self-standards (ideal, ought, undesired and forbidden selves) and related eight self-discrepancies. Four discrepancies reflect the relations between self-standards and the actual self, while, the other four reflect the relations between the same standards and the can self. The study aimed at verifying the hypothesis that apart from the perceived actualization of standards the perceived attainability of those standards are integral aspects of the structure of self-beliefs.

The main goal of this study, however, was to verify a hypothesis regarding the hierarchical organization of self-standards. It was hypothesized that the distinction between positive and negative standards is superior to the distinction between ideals and oughts. Confirmatory factor analysis (CFA) was used to test the postulated hierarchy of standards. In terms of CFA, the hypothesis claimed that the hierarchical latent variable model with the positive versus negative distinction at the higher level and the ideal versus ought distinction at the lower level of the hierarchy of self-standards fits data better than the alternative model with the opposite hierarchy of standards (i.e. with the ideal versus ought distinction at the higher level and the positive versus negative distinction at the lower level).

## Method

### Participants and Procedure

The participants were Polish students, representing a wide range of academic majors. The main part of the study was conducted via the Internet. According to the terminology proposed by Nosek et al. (2002), it was an Internet study with invited accessibility design and targeted advertising as recruitment method. In the course of the recruitment process, the researcher met groups of students during their classes and, after introducing himself and giving short oral information about the study, distributed small paper leaflets. The leaflets contained invitation to take part in the study, general information about the study, the link to the Web page, a login, a password, an individual code, and the researcher’s contact information. The students were free to take or not to take the leaflet as well as to take an additional one for a friend who could be interested in participation.

The Web application used in the study was available only for those who used the information from the leaflet. The login and password served as a protection against accidental users. The individual code allowed to ensure additional control of multiple participation of the same person (see Birnbaum 2004). A total of 1,550 leaflets inviting to take part in the study were distributed among potential participants, of whom 451 (292 women) logged in on the Web page and completed the procedure. Data from 40 participants were removed because either the content of the responses suggested that they were provided thoughtlessly or the participant explicitly expressed negative attitude towards the study (see Discussion for more details). Additional 7 records were removed due to technical server errors that occurred during the process of data collection. The final sample was composed of 404 participants (275 women) between the ages of 18 and 31 (*M* = 21.04; *SD* = 1.85).

### Measure

Self-discrepancies were measured using a newly developed computerized procedure, inspired by (though substantially more elaborate than) the computer version of Higgins’ Selves Questionnaire (Higgins et al. 1997). The procedure started with a short (approximately five-minute) practice trial, whose aim was to familiarize the participant with the formal and technical aspects of the instrument. Then the measurement of self-discrepancies itself followed. It consisted of two elements: (a) listing the descriptions of self-standards and (b) assessment of self-discrepancies. First, participants described the content of their self-standards by generating four lists. Each list consisted of 6 attributes, referring to ideal, ought, undesired, and forbidden selves, respectively. As a result, each participant provided 24 idiographically generated attributes (see Appendix for example attributes). After each list had been generated, an additional instruction asked the participant to mark those attributes that were subjectively the most important in that particular context (ideal, ought, undesired, or forbidden). The marked attributes were assumed to be the most salient content of self-standards, which was reflected in the subsequent calculation of self-discrepancy indices (see Appendix for more details).

In the second part of the procedure, the descriptions of self-standards served as a basis for the assessment of the perceived actualization of standards and their perceived attainability. For this purpose, the previously listed 24 attributes were twice presented in a random order. Firstly, to assess the discrepancies between standards and the actual self (i.e. perceived actualization of standards), participants rated the extent to which they actually possessed a given attribute. Secondly, to assess the discrepancy between standards and the can self (i.e. perceived attainability of standards), participants rated the extent to which they believed it was possible for them to acquire a given attribute.

The self-discrepancy ratings were given using electronic visual analogue scales (VAS; see Jamison et al. 2002). The VAS were presented as horizontal lines with verbal anchors labeling the ends. A small vertical slider was set by default at the middle of each line. Using the mouse, participants moved the slider along the line and set it at the appropriate point that best indicated their perceived actualization (or attainability) of a given standard. In the case of the assessment of discrepancies between standards and the actual self, the individual answered the question “To what extent are you …?” – with the previously generated 24 attributes inserted one by one in a random order. Participants indicated their perceived actualization of every standard-attribute using the VAS anchored by: *I am not like this at all* and *I am like this very much*. An analogous procedure measured discrepancies between standards and the can self. The question participants answered in this case was: “To what extent is it possible for you to become …?*”*. The appropriate VAS was anchored by: *It is completely impossible for me to become like this* and *It is definitely possible for me to become like this*. The software transformed VAS responses into discrepancy scores ranging from zero to 100, where 100 indicated the highest level of discrepancy between a given standard attribute and the actual self or the can self, respectively (for illustration of this assessment, see Appendix). The overall discrepancy was operationalized as the weighted mean of scores for 6 attributes describing a given standard. Weights were assigned depending on whether a particular attribute had been marked as “the most important” one. The ratings for the marked attributes were weighted by 2, while the ratings for the unmarked attributes were weighted by 1. The total of ratings for all six attributes multiplied by their weights was then divided by the sum of the weights. The overall procedure provided the measurement of 8 different discrepancies, as listed in Table 1. They served as observable variables in the CFA modeling.

**Table 1:** The summary of means, standard deviations, and intercorrelations for self-discrepancy scores

Self-discrepancy	M	SD	1	2	3	4	5	6	7	8
1. IA: ideal-actual	43.81	17.76	-							
2. IC: ideal-can	26.33	16.26	.69	-						
3. OA: ought-actual	38.40	17.07	.62	.47	-					
4. OC: ought-can	22.91	14.73	.49	.74	.70	-				
5. UA: undesired-actual	64.46	19.24	−.45	−.33	−.40	−.33	-			
6. UC: undesired-can	62.67	20.93	−.32	−.26	−.26	−.23	.70	-		
7. FA: forbidden-actual	58.90	20.82	−.41	−.29	−.39	−.34	.66	.47	-	
8. FC: forbidden-can	58.65	23.09	−.31	−.24	−.29	−.28	.51	.73	.73	-

All correlations are significant at *p* < .001

## Results

The main objective of the study focused on the hierarchical organization of self-standards. It was tested with the second-order CFA models. The hierarchy of self-standards was conveyed by the configuration of latent variables in the models. Self-discrepancies served as observed (input) variables in the analyses. The procedure described in the previous section provided eight self-discrepancy indices. Four out of these eight variables refer to the perceived actualization of standards (discrepancies between self-standards and the actual self), while the other four reflect the perceived attainability of the same standards (discrepancies between self-standards and the can self). Descriptive statistics for these eight variables are presented in Table 1. All correlations among self-discrepancy indices were significant though their strength varied form weak to strong. For every discrepancy involving a positive standard (i.e. the ideal self or the ought self) paired with any discrepancy involving a negative standard (i.e. the undesired self or the forbidden self) the correlations were negative and varied from weak (*r* = −.23) to moderate (*r* = −.45). In contrast, when both correlated discrepancies referred to positive or to negative standards the coefficients were positive and varied from moderate (*r* = .47) to strong (*r* = .74).

Confirmatory factor analyses were conducted using Amos 21 (Arbuckle 2009). Because of multivariate kurtosis (*c.r.* = 18.67), the asymptotic distribution-free (ADF) estimator (see Byrne 2010) was used for both models presented below. There were neither missing data nor multivariate outliers in the data set. Based on the recommendations by Kline (2005) and by Schreiber et al. (2006), the following criteria were applied to evaluate the goodness of fit: the chi-square (*χ* ^*2*^) likelihood ratio statistic, Comparative Fit Index (CFI), Tucker-Lewis Index (TLI), and the Root Mean Square Error of Approximation (RMSEA) with 90 % confidence intervals (CI). To correct the sensitivity of *χ* ^*2*^ to sample size, the ratio of *χ* ^*2*^ to *df* is also reported, with the value of the ratio lower than or equal to 2 indicating acceptable fit. CFI and TLI values higher than or equal to .95 and for the RMSEA value lower than .05 were accepted as indicative of good fit. Akaike’s Information Criterion (AIC) was used to compare nonnested models, with smaller values representing better fit (Byrne 2010; Schreiber et al. 2006).

**Fig. 1:**
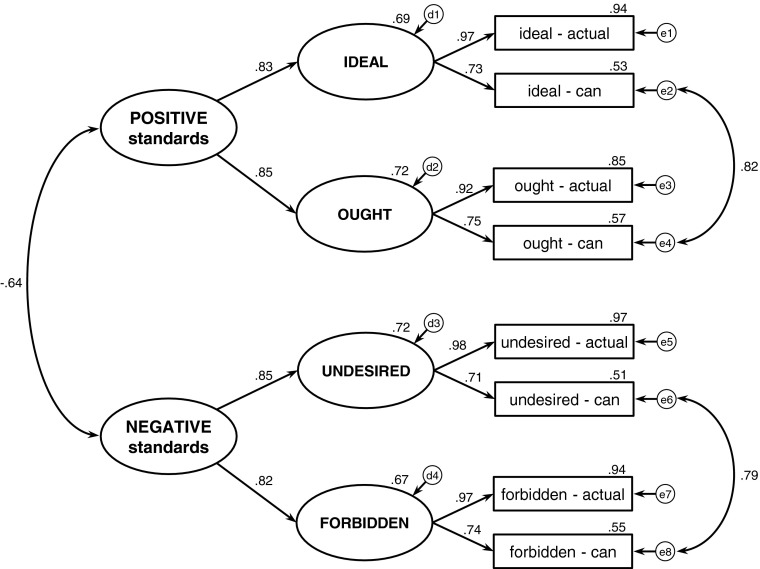
Model 1 – the hierarchical model of self-standards and related self-discrepancies. The hierarchy of self-standards is represented at the level of latent variables (*ellipses*). The observed variables (*rectangles*) are discrepancies between self-standards and the actual self/the can self. Standardized estimates are reported

It was hypothesized that the distinction between positive and negative standards is more basic than the distinction between ideals and oughts. The first model (see Fig. 1) reflects the hypothesized hierarchical structure of self-standards and related self-discrepancies. The model consists of two levels of latent variables. There are two latent variables at the higher (more general) level of the hierarchy. Each of them splits into two more detailed latent variables, which results in four variables at the lower level of the hierarchy. The distinction between positive versus negative standards can be seen at the higher level of the hierarchy, while the lower level reflects the distinction between ideals and oughts. Self-discrepancies operate at the level of observed variables.

Each of the four lower-level latent variables is loaded by a similar pair of observed variables. Each pair consists of two analogous types of self-discrepancies. The first concerns the perceived actualization of a particular standard, while the second one concerns the perceived attainability of the same standard. The pairs of observed variables differ only in the standard that is involved in the two self-discrepancies. The first pair clusters discrepancies involving the ideal self – the perceived actualization and the perceived attainability of ideals. Thus, the resulting latent variable is called “ideal.” The second pair clusters analogous ought-discrepancies, while the third and fourth pairs cluster discrepancies involving undesired and forbidden selves, respectively. Thus, the content of the latent variables from the lower level of the hierarchy is defined by both perceived actualization and perceived attainability of a particular standard. The same pattern is true in the case of all four standards: ideal, ought, undesired, and forbidden selves. The reason why a particular latent variable gets the name of a specific self-standard is that this standards is the common element of all observed variables (self-discrepancies) which load on this specific latent variable.

Moving to the higher level of the hierarchy, one can see that latent variables reflecting four standards form two more general variables. One of them clusters positive standards (both ideal and ought), while the other one clusters negative standards (both undesired and forbidden). Such a structure of latent variables clearly corresponds to the typology of self-standards as proposed in the Introduction section. This model fits data very well, which supports the main hypothesis (see Table 2, Model 1). Although the correlations between two pairs of residuals have not been predicted in the postulated model, they have a theoretical justification. The variables whose errors are correlated have similar meaning. In both cases, the errors concern the perceived attainability of standards: the attainability of positive standards in the case of correlation between e2 and e4 and the attainability of negative standards in the case of correlation between e6 and e8.

**Table 2:** Fit indices for the CFA models

Model	*χ* ^2^	df	p	*χ* ^2^/df	CFI	TLI	RMSEA (90 % CI)	AIC
Model 1	11.19	13	.595	.861	1.00	1.00	.001 (.001 – .043)	57.19
Model 2	101.23	13	.000	7.787	.867	.713	.130 (.107 – .154)	147.23

**Fig. 2:**
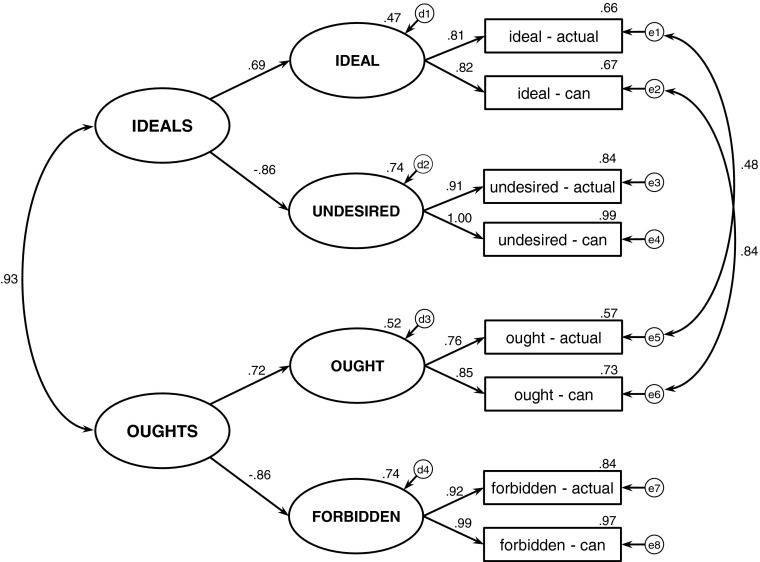
Model 2 – the alternative version of the hierarchical model. Standardized estimates are reported

In order to strengthen the verification of the proposed hierarchy of standards, an alternative model was tested (see Fig. 2). The modified model defines the hierarchy of standards in the opposite direction, making the ideal versus ought distinction superior to the distinction between positive and negative standards. As a result, both ideals and oughts split into their positive and negative aspects – in contrast to the previous model, where both positive and negative standards split into their ideal and ought aspects. Similar to the first model, two pairs of correlated residuals were added (according to the modification indices). Even after allowing for the correlated residuals the alternative model does not fit the data (see Table 2, Model 2), which supports the postulated hierarchy of standards.

## Discussion

Assuming that self-knowledge plays an important role in the processes of self-regulation, the present study focused on the structure of self-beliefs. The aim of the study was to construct and test a structural model of self-beliefs that would later be useful as background for analyses of self-regulation processes. The starting point for building such a model was the typology of self-standards, which served as a basis for distinguishing eight self-discrepancies. Two types of positive self-standards were distinguished (the ideal self and the ought self) along with the analogous two types of negative self-standards (the undesired self and the forbidden self). The content of each standard was assessed in terms of their perceived actualization (discrepancy between a self-standard and the actual self) and their perceived attainability (discrepancy between a self-standard and the can self). The results confirmed the hypothesized structural model of self-knowledge, which can be summarized by two general points:

- There are two levels in the hierarchy of self standards, with the distinction between positive and negative standards being more basic than the distinction between ideals and oughts.
- Apart from discrepancies between standards and the actual self (perceived actualization of standards), discrepancies between standards and the can self (perceived attainability of standards) are integral aspects of the structure of self-beliefs.

### The Hierarchy of Self-Standards

The main implication of the present study refers to the hierarchy of self-standards. The obtained data fit the postulated model, in which the higher-level (i.e., more basic) distinction between negative and positive standards splits into lower-level distinctions between ideals and oughts. The alternative model, assuming the opposite direction in the hierarchy of standards (with the distinction between ideals and oughts as more basic) does not fit the data. Looking for support for the proposed hierarchy in the structure of self-knowledge, some clear analogies in the broader theoretical context of evaluations and attitudes can be found. Starting from Osgood’s early work, we can see the positive–negative distinction as the most fundamental dimension of meaning (Osgood et al. 1957). This idea is further clearly and explicitly present in conceptualizations of attitudes (e.g., Cacioppo and Berntson 1994; Eagly and Chaiken 2007). If positive vs. negative evaluation is the basic dimension in attitudes towards external objects, the same should also be observed in attitudes toward oneself. This is what the present study shows. The structure of evaluating one’s own attributes clearly corresponds to the general structure of meaning in attitude formation.

The idea that this positive–negative dichotomy is a basic distinction in the structure of self-knowledge is also consistent with some neuropsychological data. Research by Davidson and collaborators (e.g., Buss et al. 2003; Silva et al. 2002; Tomarken et al. 1992) showed a functional asymmetry in the activation of the prefrontal cortex when approach vs. avoidance behavioral processes operate. Connected with this are differences at the level of positive and negative affect. The analysis of EEG data revealed connections between the activation of the right prefrontal cortex and negative affect, exposure to negative stimuli, and a behavioral tendency to avoid and withdraw. On the other hand, the connections have been found between the activation of the left prefrontal cortex and positive affect, exposure to positive stimuli and behavioral tendency to approach and engage (e.g., Buss et al. 2003; Tomarken et al. 1992). Referring to these results, Carver (2001) suggested that approach and avoidance processes (as described in his theory of self-regulation) are rooted in and governed by distinct neural systems. Assuming that approach processes engage positive standards and avoidance processes engage negative standards, we can speculate about the distinctive connections between negative standards and right prefrontal cortex and between positive standards and left prefrontal cortex. Consequently, if the two types of standards have independent neurological correlates, the distinction between positive and negative aspects of self-knowledge should be seen at the very basic level in the structural models of self-knowledge. This is exactly what the model proposed in the present study reflects.

What are the implications of this model to current theories describing the regulatory functions of self-standards? Let us consider two prominent theories: (a) Higgins’ (1997) regulatory focus theory as well as (b) Carver and Scheier’s (1998) model of self-regulating feedback systems. In Higgins’ (1997) theorizing, the most basic distinction is the one between ideals and oughts, which is the starting point for describing two modes of self-regulation – promotion and prevention regulatory foci. By contrast, in Carver and Scheier’s (1998) model the most basic processes of self-regulation are described in terms of approach and avoidance systems, with positive and negative standards as reference values for the two systems, respectively. The hierarchical models tested in this study suggest that it is Carver and Scheier’s rather than Higgins’ model that more accurately reflects the basic distinctions in self-knowledge (at least when self-knowledge is analyzed for the sake of better understanding of self-regulatory processes). This does not mean, however, that Higgins’ (1987) model is no longer valid. The CFA confirms that the ideal self is distinguishable from the ought self and, likewise – that the undesired (i.e., un-ideal) self is distinguishable from the forbidden (i.e., un-ought) self. Yet, the ideal-ought dichotomy seems to be inferior to the broader positive–negative dichotomy. Approaching positive standards and avoiding negative standards – these are the two basic modes of behavior regulation. Still, there are two slightly different modes of both approach and avoidance. The general behavioral tendency of approaching positives diversifies into two content-specific modes: approaching ideals and approaching oughts. Similarly, the general behavioral tendency of avoiding negatives diversifies into two content-specific modes: avoiding the undesired self and avoiding the forbidden self.

This idea of hierarchical organization of self-standards (and the related regulatory processes) is very similar to the two-level model recently proposed by Strauman and Wilson (2010). They distinguished between biobehavioral and social cognitive systems for approach and avoidance. The basic, biobehavioral level refers to temperamental tendencies usually described in terms of behavioral activation (BAS) and behavioral inhibition (BIS) systems. The lower, more specific level refers to strategies for approach and avoidance that are acquired through social learning. Promotion vs. prevention strategies, for which ideals vs. oughts serve as self-regulatory standards (Higgins 1997), were proposed as a good exemplar for this lower level. The results of the present study are consistent with this two-level model, which is especially interesting given that I did not include any data from the biobehavioral level in my analyses. In this context I would venture to hypothesize that (a) both biobehavioral and social cognitive levels, as proposed by Strauman and Wilson (2010), are reflected in self-knowledge and; (b) consequently, they can be detected by analyzing the structure of self-beliefs using a latent variable methodology.

### Perceived Attainability of Standards

The present study supports the postulate of including the perceived attainability of standards (i.e. discrepancies between self-standards and the can self) in the model of self-knowledge. Perceived attainability operates at the level of observed variables, loading on each lower-level latent variable. There are analogous pairs of observed variables, each pair reflecting both the perceived actualization of a given standard and its perceived attainability. This is true in the case of all four self-standards – the ideal self, the ought self, the undesired self, and the forbidden self. If we assume that the model represents the structural underpinnings of self-regulatory processes, this pairing of observed variables suggests that the regulatory functions of every self-standard involve two parallel cognitive processes: (a) assessing the degree to which the actual self is like the standard, and (b) assessing the degree to which a given standard is perceived as possible to be achieved in the future.

The basic idea of most theories of self-regulation is that the evaluation of the self from the perspective of standards leads to certain behavioral tendencies – namely, tendencies to move the self closer to the standard (in the case of positive standards) or to move it away from the standard (in the case of negative standards). It is also assumed that the instigation of those approach or avoidance behaviors depends mainly on the perceived discrepancy between the actual self and standards (Carver and Scheier 1998; Higgins 1987, 1997; Ogilvie 1987). The model tested in the present study suggests that it is a function of combined assessments of both the actualization and the attainability of standards.

One can imagine an individual who perceives themselves as very different from their ideals (high actual-ideal discrepancy), which might result in negative affect and reduced motivation. At the same time, however, that individual may be strongly convinced that he or she has all the capacities and resources that are required to reach this ideal, long-term goal in the future. Such a belief should enhance motivation and efforts in realizing the goal and reduce the negative affect. On the other hand, one can imagine the opposite situation, in which a person is close to his or her ideal goal, which should promote efforts to work towards that goal. Yet, if the person does not believe in his or her capabilities to reach this proximal goal, such an effect will probably not occur.

Thus, it is hypothesized here that the regulatory functions of the perceived actualization of standard is moderated by the perceived attainability of that standard. A similar idea has, in fact, already been formulated (Carver et al. 1979; Duval et al. 1992; Silvia and Duval 2001). Unfortunately, it has not resulted in making the discrepancies between self-standards and the can self stable and proper elements of self-discrepancy models. The present study provides arguments for doing so.

### Limitations and Future Research Directions

Conducting the study via the Internet turned out to be a convenient and efficient way of collecting data. Skitka and Sargis (2006) argued that filling out measures in the familiar context of one’s own home, in contrast to the unfamiliar laboratory context, allows people to respond more naturally. The motivation and involvement of those who decided to participate is then probably higher, which may substantially improve the quality and reliability of data. However, there are also significant disadvantages and limitations of Internet studies, some of them pertaining to the present study as well. First, the response rate to an Internet study is usually low and varies from 10 to 25 %, depending on the recruitment method (Birnbaum 2004; Skitka and Sargis 2006). The 30 % response rate of the present study seems to be slightly higher than average, but it still indicates that 70 % of potential participants did not log on or dropped out before finishing the study. The results, then, may not be free of the sampling bias.

Second, the high anonymity and low accountability of an Internet study may increase the risk of getting unreliable data (Skitka and Sargis 2006). The present study had to deal with this problem as well, since the data from 40 participants (8 % of those who logged on) were removed from the main analyses due to one of the following reasons: (a) the descriptions of self-standards were meaningless (e.g. “aaaaaaa,” “dddddd”); (b) they contained vulgar words, which explicitly indicated a negative attitude towards the study; (c) the VAS responses were made thoughtlessly (as indicated by clicking the “further” button without making any assessment).

Third, the obtained results should not be directly generalized beyond the student population. Generalization problems in Internet studies arise from the fact that Web users differ from non-users in terms of age, education, and socioeconomic status (Jackson et al. 2003; Suarez-Balcazar et al. 2009). This does not apply to the student population because virtually all the students are Web users. Thus, the presented model can be applied to students’ self-knowledge, regardless of the fact that the data were collected via the Internet. Nevertheless, the results should not be generalized to the broader population, particularly to older adults from low-income backgrounds and with a low level of education.

Regardless of the generalization issue, however, the obtained results may serve as an inspiration for new hypotheses. The study presented in this paper was focused solely on self-knowledge – i.e., the subjective perception of oneself and the structural relations between different aspects of this cognitive phenomenon. Although no objective indicators of behavior were measured, the theoretical models of self-regulation (e.g., Carver and Scheier 1998; Higgins 1997) allow to predict some regulatory implications of self-knowledge structure. Still, further studies are needed to address this problem directly. The regulatory functions of the can self seem to be a possibly challenging problem. How do the regulatory consequences of making the can self momentarily accessible compare with the effects of activating self-standards? Does the perceived attainability of standards directly enhance the process of goal attainment or does it rather moderate the regulatory functions of the perceived actualization of standards? Future experimental research may use the proposed structural model to investigate the dynamic processes of self-regulation.

## Appendix

**Table 3:** Example attributes of self-standards (ideal, ought, undesired, and forbidden selves) as reported by a randomly chosen 20-year-old male participant and the results of his self-discrepancy ratings, i.e., the assessment of perceived actualization and perceived attainability of each self-standard attribute.

Self-standards’ attributes	Weight assigned to the attribute depending on whether it was marked as “the most important”^a^	Perceived actualization: *To what extent are you …?* ^b,c^	Perceived attainability: *To what extent is it possible for you to become …?* ^b,c^
The ideal self			
*What kind of person would you like to be? Name the traits you would like to possess, ones that describe you as you would like to be in accordance with your dreams, wishes, and aspirations.* ^c^			
Assertive	2	56	6
Brilliant	1	45	36
Unpredictable	1	65	33
Athletic	1	77	50
Eloquent	2	49	27
Resourceful	2	46	27
The ought self			
*What kind of person should you be? Name the traits you should possess, ones that describe you as you should be in accordance with your sense of duty and responsibility.* ^c^			
Responsible	2	46	21
Consistent	1	37	30
Ambitious	1	43	32
Hard-working	2	58	50
Polite	1	34	23
Caring	2	38	9
The undesired self			
*What kind of person wouldn’t you like to be? Name the traits you would not like to possess, ones that describe you as you would not like to be.* ^c^			
Nervous	2	36	62
Overbearing	1	41	73
Gloomy	1	52	88
Lazy	2	40	82
Cynical	2	56	78
Brusque	1	54	47
The forbidden self			
*What kind of person shouldn’t you be? Name the traits you should not possess, ones that describe you as you should not be.* ^c^			
Lazy	2	40	82
Egocentric	2	32	75
Nervous	1	36	62
Unfair	1	41	74
Cynical	2	56	78
Fault-finding	1	48	67

*Note.* The table reflects a schematic presentation of the results of the individual participant. It is not, strictly speaking, a copy of the measure used in the study, which was a computerized procedure (see the Method section for details).

^a^After the list of a particular self-standard’s attributes had been generated, participants indicated those attributes that they perceived as the most important among those listed for that particular self-standard. It was possible to mark any number of attributes (up to six) or not to mark any of them. The marked attributes was assigned weight 2 while the unmarked was assigned weight 1. The weights were used for the subsequent calculation of self-discrepancy indices, which was operationalized as the weighted mean of ratings (actualization and attainability) for the six attributes of each self-standard.

^b^The perceived actualization and perceived attainability of self-standards’ attributes were rated using electronic visual scales. The ratings were then transformed by the software to a 101-point scale, ranging from 0 (perfect congruence between a given self-standard’s attribute and actual self/can self) to 100 (maximum discrepancy between a given self-standard’s attribute and the actual self/the can self).

^c^Italicized are the verbatim instructions presented to participants.

## Footnotes

1 Although the concept of possible selves (Markus and Nurius 1986) is somewhat broader than the concept of self-standards, the core of theorizing on possible selves refers to self-standards as well. Even if there are possible selves different form self-standards, every self-standard represents the category of possible selves.

2 As the *forbidden self* is the only new term among the four names of self-standards, it requires an additional short comment. I decided to use this new term instead of the term feared self for two reasons. Firstly, the literature on possible selves is not specific as to whether the feared self is the self that one believes one should not become or the self that one does not want to become. Secondly, the word *feared* has clear emotional connotations, which draws attention mainly to the emotional consequences of attaining self-standards and away from other important aspects of self-regulation. Moreover, it focuses specifically on fear, while it is not obvious that, of all emotions, fear is the most important emotional consequence of realizing a negative standard. For these reasons, the term forbidden self was chosen. It points explicitly to the imagined self that the person believes they ought not to become. It also seems to have connotations broader than only emotional.
